# Motor and cognitive outcomes in pediatric intestinal failure: A longitudinal cohort study

**DOI:** 10.1016/j.intf.2026.100383

**Published:** 2026-07-01

**Authors:** Anna Gold, Catherine Patterson, Christina Belza, Dylan Johnson, Bianca C. Bondi, Alaine Rogers, Paul W. Wales, Yaron Avitzur, Stephanie So

**Affiliations:** a Department of Psychology, The Hospital for Sick Children, 555 University Ave, Toronto, Ontario M5G 1X8, Canada; b Transplant and Regenerative Medicine Centre, The Hospital for Sick Children, 555 University Ave, Toronto, Ontario M5G 1X8, Canada; c Research Institute, The Hospital for Sick Children, 555 University Ave, Toronto, Ontario M5G 1x8, Canada; d Department of Rehabilitation Services, The Hospital for Sick Children, 555 University Ave, Toronto, Ontario M5G 1X8, Canada; e Department of Rehabilitation Services, University of Toronto, 500 University Ave, Toronto, Ontario M5G 1V7, Canada; f Department of Psychiatry and Behavioural Neurosciences, McMaster University, 1200 Main St W, Hamilton, Ontario L8N 3Z5, Canada; g Cincinnati Center of Excellence in Intestinal Rehabilitation, (CinCEIR), Cincinnati, USA; h Division of Pediatric General and Thoracic Surgery, Cincinnati Children’s Hospital Medical Center, Cincinnati, USA; i Department of Pediatrics, University of Cincinnati, Cincinnati, USA; j Division of Gastroenterology, Hepatology and Nutrition, The Hospital for Sick Children, 555 University Ave, Toronto, Ontario M5G 1X8, Canada

**Keywords:** cognition, motor skills, neurodevelopmental outcomes, pediatric intestinal failure

## Abstract

**Background:**

Improved survival in pediatric intestinal failure (IF) necessitates a focus on neurodevelopmental outcomes. Existing cross-sectional research demonstrates increased risk of developmental delay. This novel study explores longitudinal motor and cognitive outcomes, and associated risk factors, in a single cohort across infancy to early school age.

**Material and methods:**

Retrospective review of children admitted to an IF program < 4 months of age between 2011 and 2016, who completed: motor assessment between 4 and 15 months corrected age (CA), motor and cognitive assessment between 12 and 32 months CA and 4–8 years. Descriptive analysis of medical, socio-demographic variables and assessment scores. Outcomes were assessed using linear mixed methods, with multi-variable analyses to explore main effects and significant interactions with time.

**Results:**

Study population included 41 children (56% male), median (IQR) gestational age 33 (28.5, 35.5) weeks and length of first year hospitalization 209 (140.8, 255) days. Five children (12%) had central nervous system (CNS) co-morbidities. Gross motor scores were below average at pre-school time-points, with significant improvement by school age; whereas fine motor and cognitive scores significantly decreased at 26–32 months CA, compared to largely average scores at 12–15-months CA and school age. Main effects predicting poorer scores included: birth weight, early motor delay, CNS co-morbidity, auditory impairment, NEC diagnosis, surgeries, sepsis, and prolonged hospitalization.

**Conclusions:**

Children with IF demonstrate developmental vulnerably in infancy and preschool years, with promising motor and cognitive gains observed by early school age. Identification of key risk factors can guide IF teams in providing targeted therapeutic interventions, mitigating longer term developmental risks.

## Introduction

Intestinal Failure (IF) is the reduction of functional gut mass under the level required to maintain normal growth and fulfill daily energy and fluid requirements through enteral nutrition [1]. A recent consensus statement specified length of dependence on supplemental parenteral support as a minimum of 60 days (within a 74 consecutive day interval) [2]. IF results from a variety of congenital and acquired conditions including: short bowel syndrome (SBS), congenital diarrhea and enteropathy, and dysmotility disorders [3]. With improved survival rates [4] it is important for clinical teams to focus on longer term developmental outcomes, maximizing cognitive, physical, psychological, and social functioning. Children with IF experience multiple co-morbidities that increase developmental vulnerability. These include prematurity and low birth weight, prolonged early hospitalization, frequent surgeries, liver disease, sepsis, and nutritional or growth issues [5], [6], [7], [8]. Various sociodemographic variables may also impact outcomes, such as maternal language status, and presence of a sibling [9]. Recent research has improved our understanding of the impact of co-morbidities on early cognitive and motor development [5], [7], [8]. At school age, at least half of the children continue to present with delays in motor proficiency [8], [10]. Variability has been observed for intellectual abilities, with a recent systematic review reporting a range of average scores to severe developmental delay [11].

Most existing studies of pediatric IF are cross-sectional, focusing on single time points and employing heterogeneous assessment measures, limiting the ability to characterize long-term developmental trajectories. To address these limitations, the current study examines motor and cognitive outcomes longitudinally in a single cohort of children with IF from infancy through early school age, using developmentally appropriate standardized measures administered at multiple time points. This longitudinal approach allows for a nuanced understanding of the impact of medical, anthropometric, and socio-demographic factors on child development. This will facilitate the provision of targeted and individually tailored therapeutic interventions, with the goal of mitigating longer term developmental risks.

## Materials and methods

### Study Population

This longitudinal cohort study included children admitted to the multidisciplinary Intestinal Rehabilitation Program (IRP) at the Hospital for Sick Children (Toronto, Canada). Criteria for admission to the program includes: dependence on parenteral nutrition (PN) support for nutrients or hydration for a duration of > 60 days due to primary IF or after intestinal resection/loss, or a residual small intestinal length of < 25% of that expected for age. Children receive developmental assessments and treatment during their initial hospitalization by physiotherapy (PT) and occupational therapy (OT) and are referred to community developmental therapy at discharge, as appropriate. Developmental progress is monitored during out-patient visits with regular PT, OT and neuropsychology assessments.

Study inclusion criteria consisted of admission to the IRP at < 4 months of age between January 1, 2011 and December 31, 2016, with completion of at least one assessment at each of the following timepoints: infant motor assessment between 4 and 15 months corrected age (CA), gross and fine motor and cognitive assessment between 12 and 32 months CA, and fine motor and cognitive assessment between 4 and 8 years of age. Children undergoing a liver/intestinal transplant prior to their school age cognitive assessment were excluded as this is a known independent developmental risk [12], [13], [14].

### Medical and demographic characteristics

This retrospective study was approved by the Research Ethics Board at the Hospital for Sick Children, with participant / guardian consent waived. Medical and sociodemographic information was collected from hospital records. Medical variables included: sex, gestational age (GA), IF etiology and category (SBS, dysmotility, or congenital diarrhea and enteropathy), percent residual small bowel length expected for age post primary surgery, and anthropometrics at birth and school age assessment: head circumference, height and weight z-scores, based on World Health Organization criteria [15], central nervous system (CNS) co-morbidities (e.g., intraventricular hemorrhage grades 3 or 4, microcephaly, periventricular leukomalacia), visual and auditory impairments (e.g., use of corrective lenses, hearing aid), and diagnosis of cerebral palsy (CP). Within the first year of life, total number of: inpatient hospital days, surgeries, and septic episodes (as defined by positive blood cultures with a change in clinical status, typically associated with a fever at presentation) were collected. Within the first six years of life, total number of: inpatient hospital days, hospital admissions, surgeries, septic episodes; total days on PN and presence of advanced IF associated liver disease (defined by serum conjugated bilirubin level of >100 u/mol/L sustained for 2 weeks, and not related to a septic episode) were collected. The first 6 years of life was selected because it corresponds to the average age at time of initial school age assessment, so allows for the capture of longer- term metrics for the entire cohort. Self-declared sociodemographic data included: annual household income (Canadian $, adjusted for 2024), immigrant status, level of maternal education, presence of a sibling at home, and distance from hospital (kilometers).

### Motor and Cognitive Assessments

Results of standardized developmental assessments were extracted from hospital records. Measures included: Prechtl’s Assessment of General Movements (GMA) [16], Alberta Infant Motor Scale (AIMS) [17], Mullen Scales of Early Learning 2nd Edition (MSEL) [18], Peabody Developmental Motor Scale 2nd Edition (PDMS) [19], Beery-Buktenica Developmental Test of Visual-Motor Integration, 6th Edition (Beery VMI) [20], and Wechsler Preschool and Primary Scale of Intelligence 4th Edition (WPPSI-IV), or the Wechsler Intelligence Scale for Children, 5th Edition (WISC-V) [21], [22]. The Mullen Early Learning Composite (ELC) was used as an indicator of overall cognitive ability at 12–15 months CA and 26–32 months CA. On the WPPSI-IV and WISC-V, the full-scale intellectual quotient (FSIQ) was the domain of interest, a composite including verbal comprehension, fluid reasoning, visual spatial, processing speed and working memory abilities. Clinically these assessments represent a valid overall indicator of general cognitive abilities, based on age/developmental level. In addition, psychological diagnoses were documented. Table 1 provides details of the assessment measures and time points of administration.

**Table 1:** Summary of Assessment Measures Administered.

**Measure**	**Domain**	**Test Description**
**Birth - 4 months CA**		
Prectchl’s General Movements Assessment^16^	Infant Motor	Observation and classification of a baby’s distinct spontaneous movement patterns. Age range - birth to 20 weeks CA
**4, 8, 12–15 months CA**		
Alberta Infant Motor Scale^17^	Infant Motor	Observational tool assessing motor skills in prone, supine, sitting and standing. Provides age matched normative data represented as percentiles. Age range - 0–18 months
**12–15, 26–32 months CA**		
Mullen Scale of Early Learning^18^	Gross, Fine and Cognitive (Early Learning)	Standardized assessment to measure language, motor and perceptual abilities. Provides age matched normative data represented as T-scores. Age range – 0–68 months
**4 – 5 years of age**		
Peabody Developmental Motor Scale- Second Edition^19^	Gross and Fine Motor	Standardized assessment to measure gross motor quotient and fine motor quotient, based on scores across various subtests assessing: Stationary, Locomotion, Object Manipulation, Grasping, and Visual Motor Integration. Provides age matched normative data represented as standard scores. Age range – 0–5:11 years
**6–8 years of age**		
Beery-Buktenica Developmental Test of Visual-Motor Integration -Sixth edition^20^	Fine Motor	A shape copying task where the child is required to reproduce a series of single and interconnecting designs, provides an estimate of visual motor integration. Provides age matched normative data represented as standard scores. Age range - 2–100 years
Wechsler Pre-School and Primary Scales of Intelligence – IV^21^	Cognitive	An estimate of overall intellectual functioning (Full-Scale IQ) based on tasks in subcomponent domains (verbal reasoning, perceptual reasoning, working memory, and processing speed). Provides age matched normative data represented as standard scores. Age range - 2:6–7:3 years
Wechsler Intelligence Scale for Children –Fifth Edition^22^	Cognitive	An estimate of overall intellectual functioning (Full-Scale IQ) based on tasks in subcomponent domains (verbal comprehension, visual-spatial abilities, fluid reasoning, processing speed, and working memory). Provides age matched normative data represented as standard scores. Age range - 6–16:11 years

Legend: CA- corrected age, IQ- intellectual quotient

### Statistical Analyses

All analyses were conducted in RStudio 2024.04.2. Participant characteristics were described with median and interquartile range (IQR) for continuous variables and frequencies and proportions for categorical variables. Standard scores on developmental assessments were summarized with means and standard deviation (SD). Associations between standard scores and medical and demographic data were examined using Spearmann correlation coefficients or Mann Whitney U tests. Three separate generalized linear mixed-effects models were fitted, with gross motor, fine motor, and cognition as dependent variables. Timepoint of assessment was included as a fixed effect, and participant ID was included as a random intercept to account for within-subject variability. Pairwise comparisons of timepoints were conducted using estimated marginal means to assess differences in developmental scores across assessments. Multi-variable analyses further examined main effects and significant interactions with time.

## Results

### Study Cohort

74 children were admitted to the IRP program at < 4 months of age between the study period, with 41 completing at least one developmental assessment at each of the 3 identified timepoints. The remaining 33 children were not included due to: deceased [3], transferred to another centre [5], graduated out of the IRP in the first year [7], transplanted [1], and incomplete assessments [17]. The majority of the children with incomplete assessments were missing the school age assessment (due to not attending follow up appointments when requested, and/or the COVID−19 pandemic restrictions for in-person assessments).

### Demographic, Medical, and Developmental Variables

Table 2 provides a detailed summary of the medical and sociodemographic characteristics of the sample along with categorical results on early infant motor assessments including Prechtl’s GMA and the AIMS. Over half of the sample was male (56%), with a median GA of 33 (28.5, 35.5) weeks. The main IF category was SBS (71%), with almost half (44%) having a NEC diagnosis, and median length of time on PN between birth to 6 years of age was 277 (187, 1958) days. It is worth noting that the group of children excluded due to incomplete assessments had comparable medical demographics to the study group (59% male, median GA of 32 [25] weeks, 41% NEC diagnosis).

**Table 2:** Medical and demographic characteristics and early motor outcomes of study participants (n = 41).

**Characteristics**	**n (%)**	**Median (IQR)**
Sex; Male	23 (56)	
Gestational age, weeks		33 (28.5, 35.5)
Prematurity Categories		
< 28 weeks	9 (22)	
28–31 weeks	7 (17)	
32–36 weeks	18 (46)	
37 + weeks	6 (15)	
Birth anthropometrics (z-scores)		
Head circumference (n = 40)		0.19 (−0.43, 1.25)
Weight (n = 40)		−0.13 (−0.64, 0.81)
Length (n = 38)		−0.24 (−0.49, 0.81)
School-age assessment anthropometrics (z-scores)		
Weight (n = 40)		−0.56 (−1.30, −0.003)
Length (n = 40)		−0.71 (−1.72, 0.22)
Intestinal failure etiology		
Necrotizing enterocolitis	18 (44)	
Abdominal wall defect	12(29)	
Atresia	6 (15)	
Volvulus, Meconium Ileus, Other	5 (12)	
Intestinal failure categories		
Short Bowel Syndrome	29 (71)	
Dysmotility	11 (27)	
Congenital diarrhea and enteropathy	1 (2)	
Central nervous system comorbidities	5 (12)	
Visual impairments (n = 39)	16 (41)	
Auditory impairment (n = 37)	5 (14)	
Cerebral palsy diagnosis	4 (10)	
Duration first year hospitalization, days (n = 40)		209 (140.8, 255)
Duration hospitalization (birth to 6 years), days		231 (164.8, 283.3)
Total inpatient hospitalizations (birth to 6 years)		4 (2,8)
Total first year septic episodes		1 (0,2)
Total septic episodes (birth to 6 years)		2 (0, 4.5)
Total first year surgeries (n = 40)		3 (2, 4)
Total surgeries (birth to 6 years) (n = 39)		4 (2, 5)
Small bowel length post-surgery, %		62 (22.5, 90)
History of advanced liver disease (birth to 6 years)	9 (22)	
Total time on parenteral nutrition (birth to 6 years), days		277 (187, 1958)
Household income, per year (CA$) (n = 37)		97,911 (82,755, 116,294)
Maternal education		
Some high school	3 (7)	
High school graduate	10 (25)	
Post-secondary graduate	28 (68)	
Distance from hospital (km)		53 (30, 167)
Immigrant status	13 (32)	
At least one sibling at time of school-age assessment	31 (76)	
**Early Motor Outcomes**	**n (%)**	**Median (IQR)**
Prechtl’s General Movement Assessment (0–4 months CA) (n = 31)		
Score 4- Definitely Abnormal	11 (35)	
Score 3- Mildly Abnormal	13 (42)	
Score 2- Sub-Optimal Normal	7 (23)	
Score 1- Normal	0	
Alberta Infant Motor Score (4 months CA) (n = 38)		
≤ 5th percentile	14 (37)	
> 5th to 10th percentile	8 (21)	
> 10th percentile	16 (42)	
Alberta Infant Motor Score (8 months CA) (n = 39)		
≤ 5th percentile-	16 (41)	
> 5th to 10th percentile -	9 (23)	
> 10th percentile	14 (36)	
Alberta Infant Motor Score (12–15 months CA) (n = 39)		
≤ 5th percentile	14 (36)	
> 5th to 10th percentile	1 (3)	
> 10th percentile	24 (61)	

Legend: CA= corrected age

**Fig. 1:**
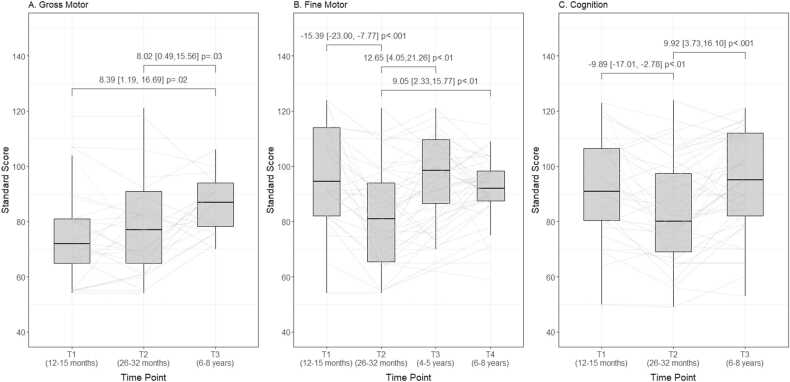
demonstrates gross motor [1a], fine motor [1b] and cognitive [1c] standard scores across identified timepoints, indicating any significant changes over time.

Between 0 and 4 months CA, over one-third (11/31) of infants assessed using Prechtl’s GMA demonstrated “definitely abnormal” scores. During the first year of life (with assessments conducted at 4, 8, and 12–15 months CA), over one-third of the infants had early motor skills falling ≤ 5th percentile on the AIMS assessment. At 12–15 months CA, although over one-third (14/39) of the children continued to fall in ≤ 5th percentile, 61% (24/39) demonstrated typically developing motor skills (≥10th percentile) **(**Table 2**).** Table 3 lists the mean standard scores on developmental measures completed between 12 months CA to 8 years of age within the three domains (gross motor, fine motor, and cognition) and indicates the timepoints used in analysis across time. Standard scores of ≤ 85 (> 1 SD below mean) were classified as having mild/moderate delays (below average) while those with a score of ≤ 70 (> 2 SD below mean) were classified as having significant delays (very below average). Scores were within age expectations, except gross motor scores at 12–15 months CA, and gross motor, fine motor, and cognitive scores at 26–32 months CA. Although there were improvements in mean scores at school age, it is important to note that ∼30–50% of the cohort still scored between 1 and 2 SD below the mean, suggesting ongoing mild delays, most notable in motor skills. Additionally, there was a large SD for scores within the mean average range, suggesting considerable variation. Fig. 1 illustrates the change in standard scores across timepoints for all domains. For gross motor skills, there was a significant improvement observed at school age compared to the preschool assessments at 12–15 months CA (p = 0.02) and 26–32 months CA (p = 0.03) (Fig. 1**a**). Fine motor standard scores fell within the average range at 12–15 months CA and school age (4–5 years and 6–8 years), with significantly poorer scores observed at 26–32 months CA when compared to all other assessment time points (p < 0.001, p < 0.01, and p < 0.01, respectively) (Fig. 1**b**). Results from cognitive assessments (Fig. 1**c**) revealed a similar profile of average scores obtained at 12–15-months CA and 6–8 years of age, with significantly lower scores observed at 26–32 months CA (p < 0.01 and p < 0.001, respectively).

**Table 3:** Developmental Outcome Measure Results.

**Study Timepoint**	**Outcome Measure (n)**	**Assessment Age**	**Mean (SD) Standard Score/Quotient**	**n (%)1SD- 2 SD below mean**	**n (%)> 2 SD below mean**
**Gross Motor**					
Time 1	Mullen Gross Motor [28]	12–15 months CA	74.89 (17.63)	9 [32]	14 [50]
Time 2	Mullen Gross Motor [40]	26–32 months CA	79.38 (20.14)	7 [18]	10 [25]
Time 3	PDMS−2 Gross motor quotient [20]	4–5 years	86.15 (10.18)	10 [50]	0 (0)
**Fine Motor**					
Time 1	Mullen Fine Motor [28]	12–15 months CA	96.82 (18.16)	7 [25]	3 [11]
Time 2	Mullen Fine Motor [40]	26–32 months CA	81.38 (21.54)	9 [23]	13 [33]
Time 3	PDMS−2 Fine motor quotient [20]	4–5 years	96.85 (16.16)	6 [30]	0 (0)
Time 4	Beery VMI [39]	6–8 years	91.38 (11.93)	4 [10]	3 [8]
**Cognitive**					
Time 1	Mullen Early Learning Composite [28]	12–15 months CA	92.64 (18.84)	7 [25]	4 [14]
Time 2	Mullen Early Learning Composite [39]	26–32 months CA	83.05 (18.03)	14 [36]	11 [28]
Time 3	WPPSI/WISC FSIQ [41]	6–8 years	93.78 (18.19)	11 [27]	3 [7]

Legend: CA- Corrected Age, PDMS- Peabody Developmental Motor Scales, VMI- Visual Motor Integration, WPPSI- Wechsler Pre-School and Primary Scales of Intelligence, WISC- Wechsler Intelligence Scale for Children FSIQ- Full Scale Intelligence Quotient

At the time of the school age assessment, psychological diagnoses were provided to 15 (37%) children in the cohort (not mutually exclusive), which included: Global Developmental Delay/Intellectual Disability 6 (15%), Learning Disability 4 (10%), Attention Deficit Hyperactivity Disorder 6 (15%) and Autism Spectrum Disorder 2 (5%).

Within the first year, poorer motor outcomes were primarily associated with factors related to prematurity, including low GA and birth weight z-score, and prolonged hospitalization. The effects of CNS co-morbidities including auditory or visual deficits or a CP diagnosis become apparent across domains at pre-school and school age assessments. Hospitalization and prematurity continue to have deleterious effects across time with additional significant risk factors across various developmental domains that include: positive caregiver immigration status, sepsis, NEC diagnosis, surgical procedures, and lower height/weight z-scores at birth or school age (See Table 4**).**

**Table 4:** Significant Correlations and Associations.

Outcome measure	Variable	R value	P value
**Gross Motor**			
AIMS* 4 months	Birth weight	.335	0.043
	Length of hospitalization first year of life	-.427	0.007
AIMS* 8 months	Gestational age	.349	0.029
	Birth weight	.322	0.048
	Length of hospitalization first year of life	-.329	0.041
Mullen Gross motor 12–15 months	Cerebral palsy diagnosis		0.015
Mullen Gross motor 26–32 months	Length of hospitalization first year of life	-.391	0.014
	Immigrant status		0.002
	Cerebral Palsy diagnosis		0.018
PDMS−2 Gross motor	Septic events first year of life	-.486	0.022
	Septic events birth to 6 years	-.508	0.015
	Prematurity < 28 weeks		0.040
**Fine Motor**			
Mullen Fine Motor 12–15 months	Length of hospitalization first year of life	-.399	0.039
	CNS co-morbidity		< 0.001
	Immigrant status		0.035
	Cerebral palsy diagnosis		0.003
	Prematurity < 32 weeks		0.037
Mullen Fine Motor 26–32 months	CNS co-morbidity		0.014
	NEC diagnosis		0.003
	Visual impairment		0.037
	Auditory impairment		0.022
	Cerebral palsy diagnosis		0.013
	Prematurity < 32 weeks		0.046
PDMS−2 Fine Motor	Sepsis birth to 6 years	-.535	0.015
Beery VMI	School age height	.383	0.016
	# hospitalizations birth to 6 years	-.322	0.046
	CNS co-morbidity		0.003
	Cerebral palsy diagnosis		0.008
**Cognitive**			
Mullen ELC 12–15 months	Septic events first year of life	-.456	0.015
	CNS co-morbidity		0.004
	NEC diagnosis		0.046
	Auditory Impairment		0.016
	Immigrant status		0.001
	Cerebral palsy diagnosis		0.024
Mullen ELC 26–32 months	Head circumference	.339	0.035
	Birth Weight	.350	0.031
	Birth Length	.361	0.028
	Length of hospitalization first year of life	-.342	0.033
	CNS co-morbidity		0.014
	Immigrant status		0.024
WISC FSIQ	Birth weight	.332	0.037
	School age weight	.381	0.015
	School age height	.399	0.011
	Surgery birth to 6 years	-.338	0.035
	CNS co-morbidity		0.002
	Visual Impairment		0.026
	Cerebral Palsy Diagnosis		0.005

Legend: AIMS- Alberta Infant Motor Scale, PDMS- Peabody Developmental Motor Scale, CNS- Central Nervous System, NEC- Necrotizing Enterocolitis, VMI- Visual Motor Integration, ELC- Early Learning Composite, WISC- Wechsler Intelligence Scale for Children, FSIQ- Full Scale Intelligence Quotient

* AIMS- converted to standard score

Table 5 outlines the factors that had a significant main effect on decreased motor or cognitive standard scores across the pre-school and school age timepoints. Poorer outcomes on infant motor assessments at 4- and 8-months CA and a “definitely abnormal” score on Prechtl’s GMA were predictive of lower gross and fine motor scores, respectively. CNS co-morbidities predicted lower scores in all three developmental domains. Having a NEC diagnosis resulted in poorer fine motor and cognitive scores, and longer length of hospitalization in the first year of life was a significant risk factor for both gross motor and cognitive development. Interestingly, length of time on PN was not significantly associated with poorer outcomes on any school age assessment domains.

**Table 5:** Significant Main Effects Across Time.

Predictor Variable	β (SE)	P Value
**Gross Motor**		
AIMS score < 10th percentile at 4 months CA	−18.26, (4.68)	< 0.001
CNS co-morbidity	−16.03, (7.69)	0.04
Length of hospitalization first year of life	−0.08 (0.03)	0.02
**Fine Motor**		
CNS co-morbidity	−23.58 (4.95)	< 0.001
NEC Diagnosis	−10.38 (4.72)	0.03
Surgeries first year of life	−3.39 (1.29)	0.01
“Definitely Abnormal” score on GMA at 0–4 months CA	−10.66 (4.30)	0.01
**Cognition**		
Auditory Impairment	−13.30 (6.23)	0.03
Birth weight	5.03 (1.99)	0.01
CNS co-morbidity	−24.63 (6.42)	< 0.001
NEC Diagnosis	−14.86 (5.74)	0.01
“Definitely Abnormal” score on GMA at 0–4 months CA	−10.94 (5.27)	0.04
Length of hospitalization first year of life	−0.06 (0.03)	0.04
Septic episodes first year of life	−3.92 (1.69)	0.02

Legend: AIMS- Alberta Infant Motor Scale, CNS- Central Nervous System, NEC- Necrotizing Enterocolitis, GMA- Prechtl’s General Movement Assessment

**Fig. 2:**
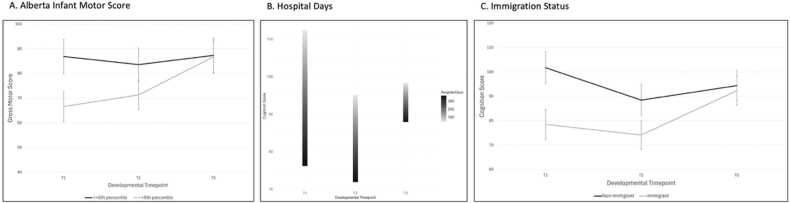
demonstrates the following factors which had a significant interaction with time: Alberta Infant Motor Score (8 months corrected age) [2a], hospital days in first year of life [2b] and immigration status [2c]. Timepoints T1: 12–15 months corrected age, T2: 26–32 months corrected age, T3: 4–8 years.

There were several significant interactions with time as illustrated in Fig. 2**.** Children with an AIMS score ≤ 5th percentile at 8 months CA had a significant improvement in their gross motor standard scores between the 26–32 months CA and school age assessment (p = 0.001) (Fig. 2**a**). At 12–15 months CA, children who spent more days in hospital during the first year of life showed significantly lower cognitive scores (β = –0.112, p = .005). By school age, the effect of hospital days had attenuated, with a significant positive interaction (β = 0.079, p = .026), indicating that the adverse impact of prolonged hospitalization diminishes over time (Fig. 2**b**). Children of immigrant caregivers had lower cognitive scores at the two preschool time points with a significant improvement between 26 and 32 months CA and school age (p < 0.001), whereas non-immigrant children performed well at 12–15 months CA, but then had a significant decrease in scores at 26–32 months CA (p = <0.001), with some recovery noted by school age (Fig. 2**c).**

## Discussion

There has been emerging evidence over recent years of the existence of developmental and cognitive challenges in pediatric IF, however most of these studies are cross sectional in nature, thus providing limited projective value. This novel longitudinal study provides more robust insight into risk factors impacting this population from birth to school age, and changes in outcome over time.

Gross motor delays throughout early childhood are consistent with the current literature [7], [8], [10], [23]. Mild delays persist in at least half of the cohort at 4–6 years of age, however, this study demonstrates a significant overall improvement in scores at this timepoint. Clinical observation suggests that infants with IF often have difficulties with transitional movements (e.g. rolling, moving in and out of sitting), as well as delays in prone skills and crawling reflected in lower scores at the 8-month assessment. Of note, lower AIMS scores in infancy can be predictive of a delay in fundamental motor skills at school age. It is encouraging to note that infants in this study who scored ≤ 5th percentile at 8 months CA (∼40% of the sample) demonstrated catch up at school age, when there is likely more opportunity to participate in structured and unstructured peer-based physical activity. It also underscores the importance of ensuring these children are referred for PT/OT therapy to help them master key developmental skills. Additionally, despite potential medical barriers such as lines, ostomies, etc., caregivers should be encouraged to provide opportunities for their children to participate in physical activities from an early age.

With respect to fine motor and cognitive results, the group demonstrated fluctuations over time, with broadly average scores obtained by school age. However, the large range of scores at school age suggest considerable within group variability, in keeping with our clinical experience. Prior research reflects this heterogeneity with some studies reporting predominantly average [24] and others noting predominantly below average [5], [10] scores. Of importance, over a third (37%) of our group were provided with one or more psychological diagnoses, warranting developmental/educational intervention. Testing cognitive abilities in isolation may not adequately capture these diagnoses. As such, it is advisable to complete comprehensive neuro/psychological assessments with children at school age to ensure important developmental needs are not overlooked.

The 26–32 months CA period appears to be a vulnerable window for fine motor and cognitive development, while gross motor scores remain low. This stage may coincide with discharge home (on or off PN) after prolonged hospitalization. Caregiver apprehension and/or medical caution may limit participation in daycare, structured programs, and peer-based play, reducing opportunities for developmental and social stimulation. In addition, various pathophysiological mechanisms may contribute to longer-term cognitive vulnerability such as metabolic disturbances [25], and sepsis [9], [26]. The importance of gut–brain interactions in pediatric IF is also poorly understood and notably under-recognized [27], [28]. Further, early childhood systemic illnesses [29], prolonged pediatric intensive care stay [25], and childhood trauma [30] are documented to also have a deleterious impact on cognitive development. The cognitive and motor catch-up observed at school age may reflect the exposure to developmentally stimulating environments. Of importance, 30–50% of the group remain in the at-risk range at school age (1–2 SD below the average), underscoring the need for ongoing participation in physical and peer-based activities, as well as close educational monitoring and advocacy.

This study demonstrates cognitive vulnerability for children with sensory (auditory impairments), anthropometric (lower birth weight), and clinical (CNS comorbidity, NEC diagnosis, greater number of early surgeries) morbidity, largely consistent with the existing literature [9], [10], illustrating the multifaceted etiology of longer-term cognitive sequelae in this population. Sociodemographic factors have been less well explored; however family stress/mental health, family structure, distance from hospital, and family income, have all been reported to impact quality of life in this population [31], [32], [33]. To our knowledge, the motor and cognitive risk posed by a positive immigrant caregiver status seen in this study is novel, and a useful clinical marker, highlighting the need for additional support and targeted clinical interventions prior to school entry for these families. Immigrant status may present as a proxy marker for more general psychosocial family-based issues including; prior trauma, loss of community and family network, cultural distance and acculturation, housing and employment insecurity, and variable access to medical, educational, and financial resources [34] - all of which can result in adaptive emotional and behavioural distress for caregivers and the wider family system. The improvement seen in cognitive function in the immigrant population at school age, may suggest the reduced burden of these factors over time and the positive impact of the school system and social integration on the child’s cognitive and language functioning.

Risk factors impacting gross motor skills are largely related to prematurity (lower GA, birth weight), CNS co-morbidity, and prolonged hospitalization during infancy and pre-school age. However, consistent with a previous study by So et al [8]. , increased septic events results in poorer gross motor skills at school age, underscoring the importance of ongoing management of septic infections in this population. Septic events also impacted school age fine motor functioning, but interestingly not cognition, which is in contrast to our prior findings [9]. The introduction of hospital wide procedures to reduce the risk of central line infections has played an important role in reducing prevalence. IRPs could also introduce other protective strategies, such as minimize the exposure to non-essential admissions/surgeries, or combine surgeries requiring general anesthesia to reduce frequency, particularly those completed within the first year of life.

Robust evidence for the potentially deleterious developmental impact of early prolonged hospitalization already exists [5], [10], with results from our cohort confirming increased time spent in hospital during infancy has long reaching effects on motor and cognitive skill development. More specifically, our analysis showed that, for every day spent in hospital during the first year of life, there is a 0.08 decrease in gross motor standard scores and a 0.06 decrease in cognitive standard scores. Given the mean length of hospital stay before 1 year of age was just over 6-months, this could represent a decrease of 16 gross motor standard score points and 12 cognitive standard score points at school age. While the complex medical care of these children may not allow early discharge home, it is important to maximize developmental, play, social and rehabilitative support within the hospital environment during those crucial early months of an infant’s life. Increased length of early hospitalization may also be associated with greater prematurity and/or illness severity, with more intensive/frequent surgical and nutritional management strategies, thus placing the child at further neurodevelopmental risk.

Primary IF centers could consider increased community outreach to develop medical expertise, to facilitate earlier discharge home, and less frequent hospital visits. This may improve accessibility of care for those families with greatest sociodemographic need. Further, providing psychoeducation to the community team on the importance of early developmental stimulation may also support timely enrollment in school/developmental programs and ongoing support for educational staff regarding medical device management within the classroom. A position statement recently published on behalf of International Intestinal Rehabilitation and Transplant Association [35] provides IRP’s with excellent evidence-based and expert-informed clinical guidance to support neurodevelopmental assessment and intervention.

There are several limitations of this study. The sample is derived from a single IRP, with a relatively small cohort at only a few timepoints, thus limiting more robust modelling and statistical analyses, as well as the generalizability of our findings. The study was a retrospective clinical study, and as such, we were unable to standardize or accurately classify the clinical developmental interventions provided to the cohort during their hospital stay or out-patient visits, thus making it challenging to delineate the potential impact on overall developmental trajectory. Given the longitudinal nature of this study, some of the participants were included in prior publications from our center which may bias the current findings. However, published studies from other centers have noted similar developmental risk factors [5], [6], [10].

## Conclusion

It is well established that children with IF are at risk of longer-term motor and cognitive delay. This study identifies specific time periods of vulnerability, primarily in infancy and pre-school years, however, shows promising gains in skills at early school age. While multiple risk factors impact development, this study uniquely indicates key factors across time (0–8 years of age) likely to result in poorer scores in various domains. These are useful clinical markers which can guide the IRP in their provision of targeted therapeutic interventions and community/educational advocacy to maximize longitudinal developmental success.

## CRediT authorship contribution statement

**Paul W Wales:** Writing – review & editing, Conceptualization. **Alaine Rogers:** Writing – review & editing, Investigation. **Bondi Bianca C:** Writing – review & editing, Visualization, Methodology. **Dylan Johnson:** Writing – review & editing, Visualization, Formal analysis, Data curation. **Christina Belza:** Writing – review & editing, Data curation, Conceptualization. **Catherine Patterson:** Writing – review & editing, Investigation. **Anna Gold:** Writing – original draft, Methodology, Investigation, Data curation, Conceptualization. **Stephanie So:** Writing – original draft, Methodology, Investigation, Data curation, Conceptualization. **Yaron Avitzur:** Writing – review & editing, Conceptualization.

## Patients/guardian consent

Waived by institutional REB.

## Ethical clearance

Ethics approval has been obtained by the institution’s REB (letter # 100081392, dated 17 March 2025)

## Funding

This research did not receive any specific grant from funding agencies in the public, commercial, or not-for-profit sectors.

## Declaration of Competing Interest

None
